# Forced Gradient Copolymer for Rational Design of Mussel-Inspired Adhesives and Dispersants

**DOI:** 10.3390/ma16010266

**Published:** 2022-12-27

**Authors:** Takehiro Fujita, Masami Shuta, Mika Mano, Shinnosuke Matsumoto, Atsushi Nagasawa, Akihiro Yamada, Masanobu Naito

**Affiliations:** 1 Data-Driven Polymer Design Group, Research and Services Division of Materials Data and Integrated System (MaDIS), National Institute for Materials Science (NIMS), Ibaraki 305-0047, Japan; 2 Program in Materials Science and Engineering, Graduate School of Pure and Applied Sciences, University of Tsukuba, Ibaraki 305-8577, Japan; 3 Oleo & Speciality Chemicals Research Lab., NOF Corporation, Hyogo 660-0095, Japan

**Keywords:** mussel-inspired polymer, adhesive, dispersant, forced gradient polymer, l-3,4-dihydroxyphenylalanine, dopamine, gallic acid, BaTiO_3_

## Abstract

In recent years, there has been considerable research into functional materials inspired by living things. Much attention has been paid to the development of adhesive materials that mimic the adhesive proteins secreted by a mussel’s foot. These mussel-inspired materials have superior adhesiveness to various adherents owing to the non-covalent interactions of their polyphenolic moieties, e.g., hydrogen bonding, electrostatic interactions, and even hydrophobic interactions. Various factors significantly affect the adhesiveness of mussel-inspired polymers, such as the molecular weight, cross-linking density, and composition ratio of the components, as well as the chemical structure of the polyphenolic adhesive moieties, such as l-3,4-dihydroxyphenylalanine (l-Dopa). However, the contributions of the position and distribution of the adhesive moiety in mussel-inspired polymers are often underestimated. In the present study, we prepared a series of mussel-inspired alkyl methacrylate copolymers by controlling the position and distribution of the adhesive moiety, which are known as “forced gradient copolymers”. We used a newly designed gallic-acid-bearing methacrylate (GMA) as the polyphenolic adhesive moiety and copolymerized it with 2-ethylhexyl methacrylate (EHMA). The resulting forced gradient adhesive copolymer of GMA and EHMA (poly(GMA-*co*-EHMA), Poly**1**) was subjected to adhesion and dispersion tests with an aluminum substrate and a BaTiO_3_ nanoparticle in organic solvents, respectively. In particular, this study aims to clarify how the monomer position and distribution of the adhesive moiety in the mussel-inspired polymer affect its adhesion and dispersion behavior on a flat metal oxide surface and spherical inorganic oxide surfaces of several tens of nanometers in diameter, respectively. Here, forced gradient copolymer Poly**1** consisted of a homopolymer moiety of EHMA (Poly**3**) and a random copolymer moiety of EHMA and GMA (Poly**4**). The composition ratio of GMA and the molecular weight were kept constant among the Poly**1** series. Simultaneous control of the molecular lengths of Poly**3** and Poly**4 ** allowed us to discuss the effects on the distribution of GMA in Poly**1**. Poly**1** exhibited apparent distribution dependency with regard to the adhesiveness and the dispersibility of BaTiO_3_. Poly**1** showed the highest adhesion strength when the composition ratio of GMA was approximately 9 mol% in the portion of the Poly**4** segment. In contrast, the block copolymer consisting of the Poly**3** segment and Poly**4** segment with only adhesive moiety **1** showed the lowest viscosity for dispersion of BaTiO_3_ nanoparticles. These results indicate that copolymers with mussel-inspired adhesive motifs require the proper design of the monomer position and distribution in Poly**1** according to the shape and characteristics of the adherend to maximize their functionality. This research will facilitate the rational design of bio-inspired adhesive materials derived from plants that outperform natural materials, and it will eventually contribute to a sustainable circular economy.

## 1. Introduction

Non-human living things have a variety of capabilities that surpass those of humans and enable them to remain active even in the harshest of environments. Such superior capabilities have inspired the creation of biomimetic materials [1,2]. For example, the adhesive capability of a mussel’s foot has been studied extensively. It is well known that catechol, which is a polyphenol, is expressed in the proteins secreted by a mussel’s foot. The byssus, which is a bundle of filaments secreted by many species of bivalve mollusk, plays an important role in underwater adhesion to target surfaces. It achieves this via non-covalent interactions, such as hydrogen bonding, electrostatic interactions, chelation, hydrophobic interactions, and oxidative cross-linking [3,4,5]. A variety of mussel-inspired adhesives that mimic a mussel’s adhesion mechanism have been developed by introducing a catechol moiety into various types of polymers, such as polypeptides [6,7], polyethylene glycol [8], polyacrylates [9,10,11,12,13,14], and polystyrene [15,16,17,18,19]. These studies have revealed that the adhesive capabilities of mussel-inspired polymers are significantly affected by their primary structures. For example, Wilker and co-workers [20] prepared poly((3,4-dihydroxystyrene)-*co*-styrene) copolymers with various molecular weights by living anionic polymerization. They found that the adhesive strength of the copolymer increases linearly as a function of its molecular weight in the range of 20,000–100,000 kDa. Similarly, Kohri and co-workers [21] reported that branched poly(*N*-(2-(2,2-dimethylbenzo-1,3-dioxol-5-yl)ethyl)-acrylamide) prepared by controlled polymerization has superior adhesion properties to similar polymers with linear structures. Ejima and co-workers [22,23,24,25] investigated the effect of the number of aromatic hydroxyl groups on the benzene rings of the adhesive moieties in such copolymers. They found that the adhesion capability increases as the number of OH groups on the polyphenol groups increases. However, it is still unclear how the position and distribution of the adhesive moieties in such polymers affect their adhesiveness, despite the fact that mussel-foot proteins have been shown to contribute profoundly to the l-3,4-dihydroxyphenylalanine (l-Dopa) sequence, not only in regulating the binding strength of l-Dopa residues but also in achieving adaptability to various surfaces [26,27].

**Scheme 1:**
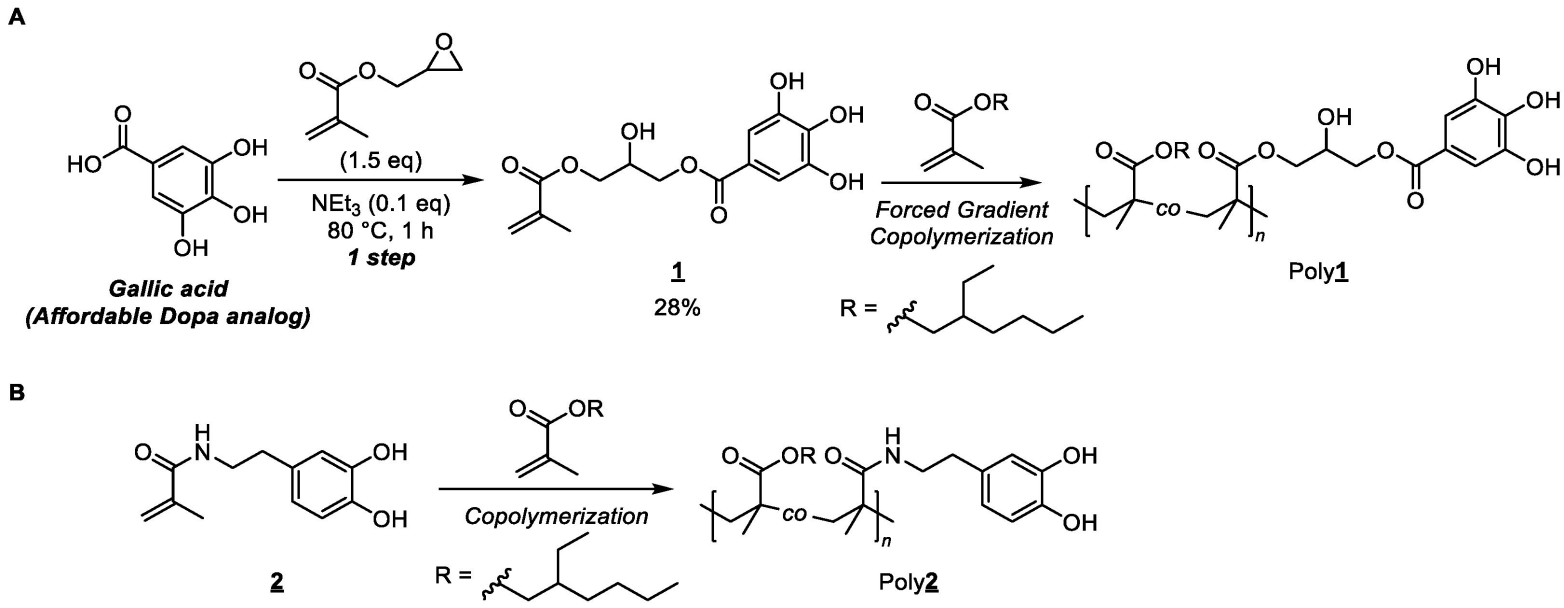
(**A**) One-pot synthesis of methacrylate-bearing gallic acid monomer **1** and preparation of poly(**1**-*co*-(EHMA) (Poly**1**, EHMA = 2-ethylhexyl methacrylate, **1** = gallic-acid-bearing methacrylate). (**B**) Structure and chemical synthesis of Poly**2**.

Within this context, we have attempted to demonstrate the effects of the monomer position and distribution of the adhesive moieties in mussel-inspired adhesive polymers on their adhesion and dispersion capabilities. To realize this, we designed a gallic-acid-bearing methacrylate monomer (**1**) that has the potential as an adhesive moiety (Scheme 1A) [12,14]. To demonstrate how the monomer position and distribution of **1** affect the adhesion and dispersion capabilities of a mussel-inspired adhesive polymer, we focused on a forced gradient copolymer (FGCP) [28,29]. An FGCP is prepared by controlling the timing of the addition of the second monomer during the polymerization of the first monomer (Scheme S1). It allows the compatibility of immiscible polymer blends or the stability of emulsions/dispersions to be systematically investigated.

In this study, to clearly characterize the position and distribution of **1** in poly(**1**-*co*-EHMA) (Poly**1**, EHMA = 2-ethylhexyl methacrylate, **1** = gallic-acid-bearing methacrylate), a series of Poly**1** copolymers with different molecular lengths of the homo- and random polymer segments was prepared (Scheme S1). The resulting forced gradient Poly**1** copolymers were subjected to adhesive tests on aluminum substrates and dispersion tests with nanoparticles of barium titanate (BaTiO_3_) perovskite oxides (50 nm in average diameter) in organic solvents (Scheme 1A). Here, the aluminum substrate and BaTiO_3_ nanoparticle were used as a flat metal oxide surface and a spherical inorganic oxide surface, respectively. The adhesion and dispersion capabilities were significantly affected by the position and distribution of **1** in Poly**1** in different ways.

## 2. Materials and Methods

### 2.1. General

All of the reactions involving the oxygen- and moisture-sensitive compounds were carried out in a dry reaction vessel under nitrogen or argon. The reaction mixtures were degassed using the freeze–pump–thaw method before the radical polymerization reactions commenced. Flash column chromatography was performed with a silica gel (60N, spherical neutral; Kanto Chemical Co., Inc., Tokyo, Japan). Analytical thin-layer chromatography was performed with a silica gel coated with fluorescent indicator F254 (silica gel 60 F254, Art 5715, 0.25 mm, Merck, Darmstadt, Germany). 

### 2.2. Materials

Triethyl silyl chloride, *N*,*N*-dimethylformamide (super-dehydrated), NaHCO_3_, tetrabutylammonium fluoride, tetrahydrofuran solution (~1 mol/L), acetic acid, and BaTiO_3_ were purchased from FUJIFILM Wako Pure Chemical Co.(Osaka, Japan)., and they were used as received. Imidazole was purchased from Tokyo Chemical Industry Co., Ltd (Tokyo, Japan)., and it was used as received. 1,2-Dichloroethane (super-dehydrated) was purchased from Kanto Chemical Co., Inc., and it was used as received. 2-Ethylhexyl methacrylate was purchased from FUJIFILM Wako Pure Chemical Co., and it was passed through an A_2_O_3_ column before use to remove the radical inhibitor. 2-Cyano-2-propyl dodecyl trithiocarbonate was purchased from Sigma-Aldrich Co., and it was used as received. 2,2′-Azobisisobutyronitrile was purchased from FUJIFILM Wako Pure Chemical Co., and it was recrystallized from methanol before use. **1** was provided by NOF CORPORATION (Hyogo, Japan).

### 2.3. Preparation of the Mussel-Inspired Forced Gradient Copolymers

To investigate the forced gradient adhesive moiety in adhesive polymers, we designed gallic-acid-bearing methacrylate monomer **1** that has the potential as an adhesive moiety (Scheme 1A). A galloyl group with three phenolic hydroxyl groups is expected to show greater adhesiveness than a catechol group with two hydroxyl groups (such as in l-Dopa), owing to the greater number of vicinal hydroxyl groups in the benzene ring [22,23,24,25]. From the viewpoint of practical application, gallic acid is more affordable than l-Dopa and its derivatives.

**Figure 1:**
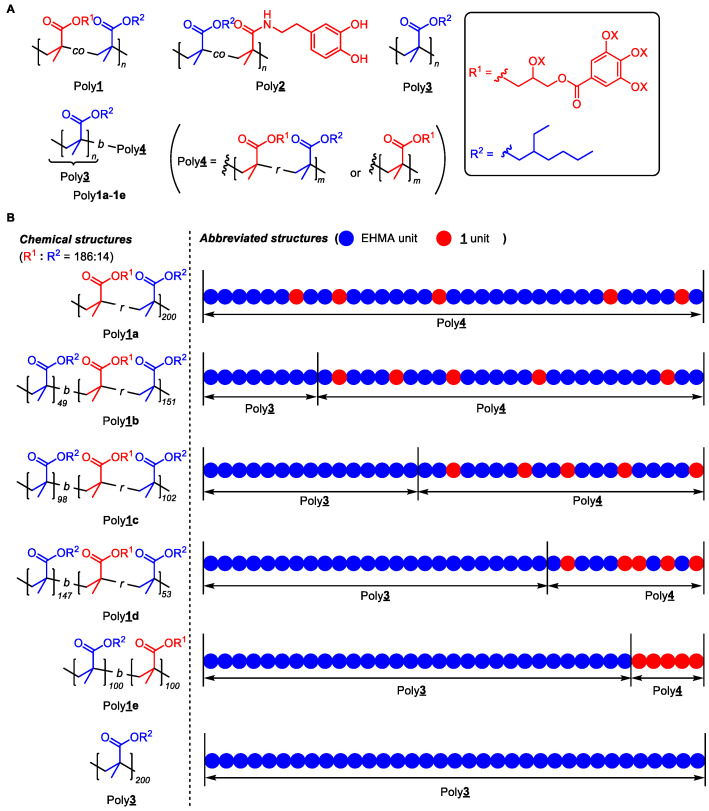
(**A**) Summary of the chemical structures of the polymers in this work. Poly**3** is a homopolymer domain of EHMA. Poly**4** is a random copolymer domain of EHMA and **1** in Poly**1a**–**1e**. (**B**) Chemical and abbreviated structures of synthesized copolymers Poly**1a**–**1e**.

In a previous study, we reported the rational design of a mussel-inspired adhesive comprising a dopamine-functionalized copolymer (Poly**2**). We varied the alkyl-chain lengths/structural isomers of the alkyl methacrylate comonomers and their ratio with dopamine-functionalized methacrylamide **2** (Scheme 1B) [14]. The resulting EHMA-based adhesive copolymer with 8 mol% dopamine exhibited tough, strong, and ductile adhesive properties. The relatively long and branched alkyl chain of EHMA is also expected to prevent oxidation of the catechol unit through hydrophobic interactions [30]. Here, we designed a gallic-acid-functionalized adhesive polymer (Poly**1**) based on Poly**2** because gallic acid, which has three hydroxyl groups, confers greater adhesiveness than l-Dopa, which has two hydroxyl groups [22,23,24,25]. Poly**1** copolymers were synthesized from EHMA and **1** by varying the position and distribution of **1** (Figure 1).

We prepared a series of gallic-acid-bearing Poly**1** copolymers by reversible addition–fragmentation chain-transfer polymerization. The resulting Poly**1** copolymers were characterized by proton nuclear magnetic resonance (^1^H NMR) spectroscopy and gel permeation chromatography (GPC). The detailed procedures used in the present study are described in the Supporting Information, and the characteristics of the copolymers are summarized in Table 1. In the Poly**1a**–**1e** copolymers, the ratio of **1** to EHMA and the degree of polymerization (*D*_p_) were standardized to 7:93 and 200, respectively. Here, Poly**1a** and Poly**1e** correspond to random and diblock copolymers of EHMA and **1**, respectively. The Poly**1b**–**1d** FGCPs comprised two domains: a homopolymer domain of EHMA (Poly**3**) and a random copolymer domain of EHMA and **1** (Poly**4**). Here, *D*_p_ of the Poly**3** segment in Poly**1b**–**1d** was varied (49, 98, and 147). It is noteworthy that, in this system, the composition ratio of **1** in the Poly**4** domain can be manipulated by tuning *D*_p_ of the Poly**3** domain. This allows a systematic evaluation of the adhesive capability of **1** regarding the monomer position and distribution of adhesive moiety **1** in the Poly**1** series.

### 2.4. Adhesion Test

We evaluated the adhesive strengths of the Poly**1** copolymers by butt tensile tests using a centrifugal adhesion test analyzer (Supplementary Materials, Figure S2A) (LUMiFrac; LUM GmbH, Germany). Each Poly**1** copolymer was coated on an aluminum butt at a coverage rate of 9.4 mg·mm^−2^, and the coated area was pre-cured at 60 °C to prevent unexpected void formation. The resulting Poly**1**-coated aluminum butt was placed on an aluminum plate, and the specimen was then cured at 80 or 120 °C for 1 h (Figure S1). From five to eight samples were loaded simultaneously into the measuring chamber (Figure S2B). A centrifugal force was applied to each test specimen at 5 N·s^−1^ and ambient temperature. The adhesive strength (MPa) was determined from the adhesive force (N) when bonding failure occurred divided by the area (mm^2^) of Poly**1** applied to the aluminum butt specimen (Table 1).

### 2.5. Dispersion Test

#### 2.5.1. Dispersion Test Evaluated by the Naked Eye

A mixture comprising BaTiO_3_ nanoparticles (50 mg, 0.21 mmol), Poly**1** (25 mg, ~5.0 × 10^−4^ mmol), and chloroform (5 mL) was sonicated for 60 min, and the resulting mixture was stirred at room temperature. The ability of the Poly**1** copolymers to act as dispersants was evaluated by the naked eye.

#### 2.5.2. Dispersion Test Evaluated Using a Rheometer 

A mixture comprising BaTiO_3_ nanoparticles (10 g, 43 mmol), Poly**1** (10 mg, ~2.0 × 10^−4^ mmol), and 1,1,2,2-tetrachloroethane (7.5 mL) was stirred at a shear rate of 1000 s^−1^, and the viscosity of the resulting mixture was monitored at a shear rate of 10–100 s^−1^ at room temperature by a rheometer (MCR302; Anton-Paar, Austria).

## 3. Results

### 3.1. Effect of the Monomer Position on the Adhesiveness

First, to confirm the ability of gallic-acid-functionalized methacrylate monomer **1** to act as an adhesive moiety, we carried out butt tensile tests on Poly**1a**, Poly**2**, and Poly**3** using a centrifugal adhesion test analyzer. Poly**2** was prepared by free-radical copolymerization of **2** and EHMA according to our previous report so that molar ratio of **2** in the copolymer was 8 mol% (see the Supporting Information) [15]. Poly**3**, which did not contain **1**, was used as a model polymer. Consequently, Poly**1a** exhibited similar adhesive strength to Poly**2** (7.9 and 8.8 MPa, respectively). In contrast, Poly**3** showed relatively low adhesive strength (2.8 MPa), suggesting that **1** acted as an adhesive moiety in a similar manner to **2**. Furthermore, Poly**1a** and Poly**2**, which both contained polyphenolic adhesive moieties, exhibited cohesive failure resulting from strong interactions with the oxidized aluminum surface (Figure S3). However, Poly**3** exhibited surface failure, probably owing to the lower adhesiveness than the adherend. Next, we subjected Poly**1** copolymers comprising **1** at various positions and with different distributions (Poly**1b**–**1e**) to butt adhesive tests. Note that Poly**1b**–**1d** consisted of two domains: Poly**3** comprising a homopolymer of EHMA and Poly**4** comprising a random copolymer of EHMA and **1**. The *D*_p_ values and composition ratios (mol%) of **1** were almost identical among the Poly**1** copolymers, whereas the ratios of *D*_p_ between Poly**3** and Poly**4** varied. The tensile strength values of the copolymers in megapascals are listed in Table 2.

**Figure 2:**
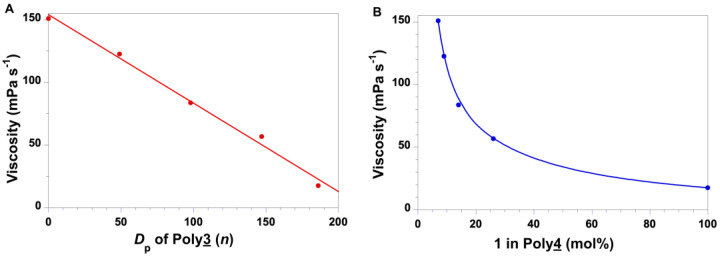
Relationships between the adhesive strength (MPa) and the (**A**) degree of polymerization (*D*_p_) of Poly**3** (*n*) and (**B**) ratio of **1** in Poly**4** (mol%).

To further clarify the effects of the position and distribution of **1** on the adhesiveness of Poly**1**, we plotted the adhesive strength versus *D*_p_ of the Poly**3** domain (Figure 2A) and the adhesive strength versus the ratio of **1** in the Poly**4** domain (mol%, Figure 2B). The curve shown in Figure 2A is rather gradual and monomodal, whereas that in Figure 2B is steep, with a maximum of 9 mol%. These results provide important insights into the effects of the monomer position and distribution on adhesion. The fact that the adhesive strength changed by only 8–10 MPa, even when the *D*_p_ value varied from 0 to 50, indicates that a non-adhesive Poly**3** domain at one end of the Poly**1** copolymer did not have a marked effect on its adhesive performance, even if that domain occupied half of its total length. In contrast, the adhesive strength clearly indicated the dependency on the composition ratio of **1** in the Poly**4** domain of Poly**1**. The adhesive strength of Poly**1** reached a maximum when it contained 9 mol% **1**, but it decreased sharply when the ratio of **1** exceeded that level. In a previous study, we found that the adhesive strength of Poly**2** depends on the composition ratio of **2**, and it reaches a maximum when the ratio is approximately 8 mol%. This arises from the balance between the polymer chain mobility and the adhesiveness conferred by the polyphenolic adhesive moiety dopamine. Considering that the same comonomer, EHMA, was used in Poly**1**, we believe that a similar phenomenon occurred in Poly**1**. Overall, the results of the adhesion tests on the forced gradient Poly**1** copolymers indicated that in Poly**4**, the maximum adhesiveness was more dependent on the ratio of **1** than the domain length of non-adhesive Poly**3**.

### 3.2. Effect of the Monomer Position on the Dispersity

**Figure 3:**
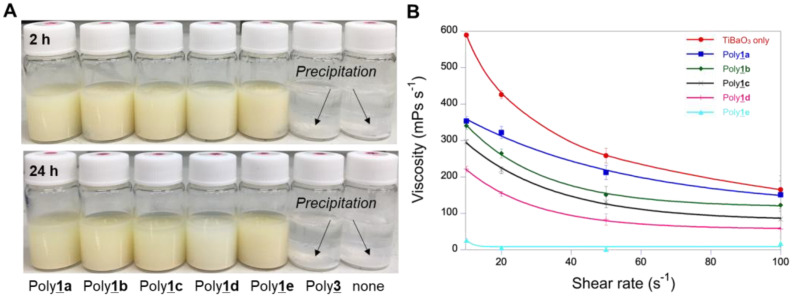
(**A**) Photographs of BaTiO_3_ dispersed in a chloroform solution in the presence of Poly**1a**–**1e**. (**B**) Relationships between the shear rate and the viscosity in dispersions comprising the copolymers, BaTiO_3_, and 1,1,2,2-tetrachloroethane.

With the miniaturization of commercial electronic products and devices, the size of electronic components has become smaller than ever. BaTiO_3_ is widely used in electronic components, such as multilayer ceramic capacitors (MLCCs), owing to its high dielectric constant [30,31]. MLCCs with even higher capacitance, smaller size, and higher reliability are required. Reducing the thickness and grain size of the dielectric layer leads to an increase in the capacitance-to-volume ratio, but such measures lead to an increase in the electric-field strength applied to the dielectric layer. Therefore, it is important to improve the dispersibility of the dielectric ceramic nanoparticles and make a low-viscosity slurry of the ceramic nanoparticles to fabricate defect-free ceramic dielectric layers. In general, MLCCs are manufactured as follows. Powdered dielectric ceramics, mainly BaTiO_3_, are dispersed in aqueous/organic solvents to obtain a slurry, which is coated on a carrier film to prepare a green sheet (raw sheet). Here, the choice of the dispersant is crucial to obtain a low-viscosity slurry of ceramic nanoparticles because a multilayer structure is formed by screen printing the inner electrode patterns on the green sheet. Eventually, the stack is sintered at 1000–1400 °C. Various types of polymer dispersants are used for the dispersion of functional inorganic/organic nanoparticles [32]. Polymeric dispersants consist of a functional group that serves as an anchor and a soluble polymer chain. For a polymeric dispersant to adsorb on inorganic oxide (nano)particles, the anchor groups must be able to strongly adsorb to the (nano)particles. Much effort has been made to find suitable polymers for this purpose. Examples of typical functional groups for this purpose are amine, ammonium, and quaternary ammonium, carboxylic acid, sulfonic acid, and phosphoric acid groups [33,34,35,36]. l-Dopa has also been introduced at the terminus of polymer chains as the anchoring moiety, leading to a highly stable iron-oxide-nanoparticle colloidal suspension [37]. However, the position of the anchoring moiety in such polymer dispersants to create l-Dopa-functionalized polymers that are suitable as dispersants remains unclear. In the present study, we demonstrated the effects of the monomer position and distribution of **1** in Poly**1** on the dispersibility of BaTiO_3_ nanoparticles in organic solvents. In brief, inorganic BaTiO_3_ nanoparticles (averaged diameter 50 nm) were added to chloroform to produce a dispersion with a BaTiO_3_ concentration of 8.4 × 10^−2^ mol·L^−1^. This dispersion was then sonicated for 60 min. The resulting suspension of BaTiO_3_ particles immediately produced a precipitate (Figure 3A). However, when 2.5 mL of a chloroform solution of Poly**1a**–**1e** (~2 × 10^−4^ mol·L^−1^) was added to the BaTiO_3_ suspension (2.5 mL) and the resulting mixture was successively sonicated for 60 min, the sediment-prone suspension of BaTiO_3_ particles turned into a stable dispersion, which could be stored for more than 24 h. Unlike Poly**1a**–**1e**, Poly**3** did not exhibit any dispensability with regard to BaTiO_3_ nanoparticles using the same procedure. To quantitatively evaluate the dispersibility of Poly**1** with regard to BaTiO_3_ nanoparticles, the viscosity of the dispersion of BaTiO_3_ was evaluated using a rheometer. First, the BaTiO_3_ nanoparticles were dispersed in 1,1,2,2-tetrachloroethane (5.7 mol·L^−1^) at a shear rate of 1000 s^−1^, and the viscosity of the resulting dispersion was monitored at shear rates of 10–100 s^−1^ (Figure 3B). The viscosity of the dispersion decreased exponentially from 589 to 165 Pa·s^−1^ as the shear rate increased from 10 to 100 s^−1^. However, a stable dispersion of BaTiO_3_ nanoparticles with a significantly lower viscosity was produced following the addition of ~2.0 × 10^−4^ mmol of Poly**1a**–**1e**. Moreover, it was obvious that the degree by which the viscosity decreased depended on the monomer sequence of the Poly**1** copolymer used.

**Figure 4:**
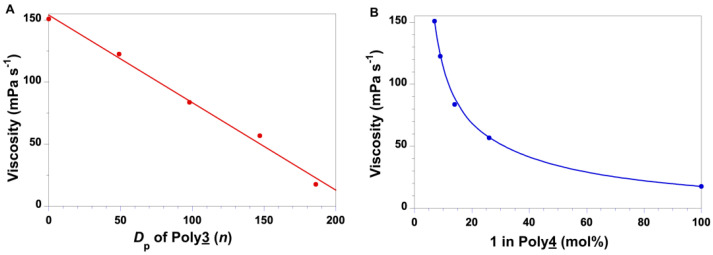
Relationships between the viscosity (mPa s^−1^) and (**A**) *D*_p_ of Poly**3** (*n*) and (**B**) the composition ratio of **1** in the Poly**4** domain of Poly**1** (mol%) at a shear rate of 100 s^−1^.

To further clarify the effect of the monomer position of Poly**1** on the dispersibility of BaTiO_3_ at a shear rate of 100 s^−1^, we plotted the viscosity versus *D*_p_ of the domain of Poly**3** (*n*) and the viscosity versus the composition ratio (mol%) of **1** in the domain of Poly**4** in Poly**1** (Figure 4). To our surprise, the viscosity behavior was completely different from that of the adhesive: the viscosity decreased linearly with *D*_p_, whereas it decreased exponentially with the composition ratio of **1** in the Poly**4** domain of Poly**1**. These results suggest that the adhesive moiety **1** should preferably be concentrated at one end of Poly**1**, and a longer Poly**3** domain contributes to better dispersion.

### 3.3. Discussion

**Figure 5:**
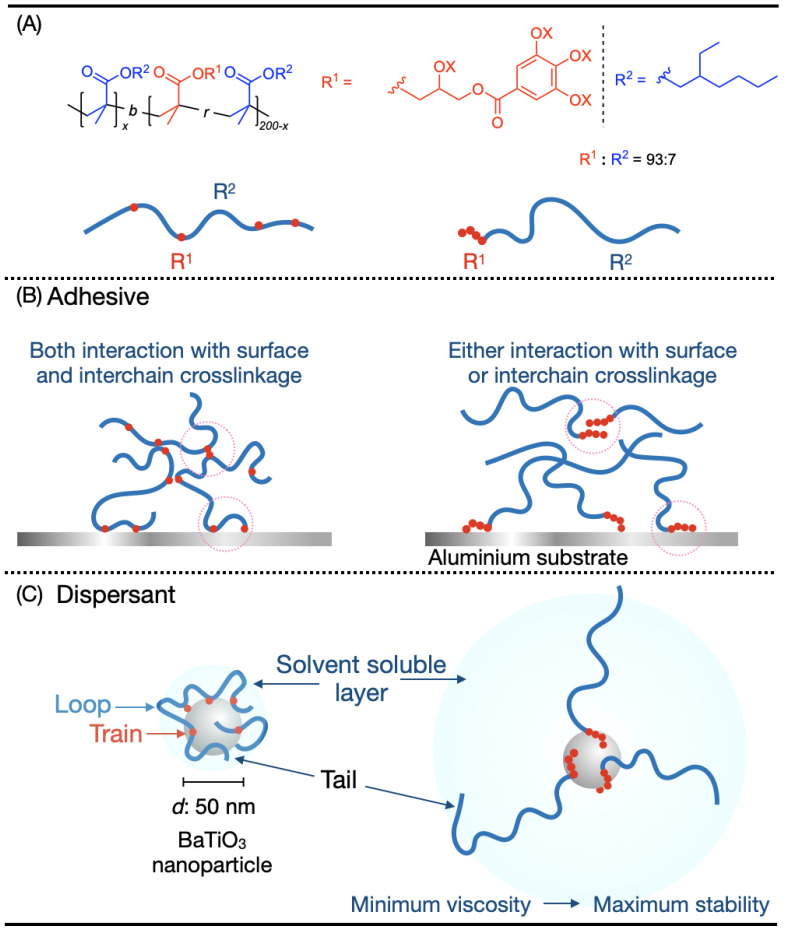
(**A**) Abbreviated structures of the copolymers. (**B**) Model structure showing copolymers bonding to aluminum plates. (**C**) Model structure showing copolymers adsorbing on a BaTiO_3_ nanoparticle.

It is worth considering why the monomer position and distribution of Poly**1** affected adhesion and dispersion in different ways. In the foot proteins secreted by mussels, the sequence of l-Dopa is known to be important for the adhesion ability [38]. However, despite the numerous reports of adhesive polymers inspired by mussel proteins, the relationship between the positions of the adhesive moieties, such as l-Dopa, and the adhesive properties has rarely been explored. We used the Poly**2** series to identify an appropriate polymer design that maximizes the adhesive strength by systematically varying the alkyl-chain length of the methyl alkylate and the composition ratio of the l-Dopa monomer [14]. The maximum adhesive strength of Poly**2** was achieved when the composition ratio of the l-Dopa monomer was approximately 8 mol% in EHMA as methyl alkylate. In addition, we found that the ductility of Poly**2** was maximized in this combination, resulting in high adhesive strength. Given the chemical structure of Poly**1**, the Poly**4** domain can be regarded as an analog of Poly**2**. Therefore, it is reasonable that the adhesive strength was maximized when the composition ratio of **1** in Poly**4** was approximately 9 mol%, probably because of an appropriate combination of adhesive strength and ductility of the Poly**4** domain (Figure 2B). In addition, when **1** is evenly located in Poly**1**, the adhesive monomer **1** not only contributes to adhesion to the adherend, but it also acts as a non-covalent cross-linkage among the gallic acid moieties (**1**), which may also contribute to the cohesion force. Indeed, the Poly**3** domain without adhesive moiety **1** was found to have a negligible effect on the adhesive ability up to *D*_p_ of approximately 100 (Figure 2A). However, when the length of the Poly**3** domain exceeds *D*_p_ = 100, adhesive monomer **1** is unevenly distributed at the end of poly**1**, making it difficult to cross-link the polymer chains by non-covalent bonds between adhesive monomers. Consequently, the cohesive force does not act on the entire adhesive layer, which is thought to lower the adhesive strength (Figure 5B). 

When Poly**1** was used as a dispersant for inorganic oxide nanoparticles, the adhesive Poly**4** segment as an anchor moiety should strongly adsorb to the nanoparticle surface, while the soluble polymer moiety should be positioned to maximize the entropy. Considering that the viscosity of the dispersion of BaTiO_3_ nanoparticles linearly decreased as a function of *D*_p_ of Poly**3**, in the dispersion mechanism of the Poly**1** series, adhesive moiety **1** in the Poly**4** segment adsorbed to BaTiO_3_, and the remaining Poly**3** at one end is thought to determine the dispersibility (Figure 4A). Figure 5C suggests a more detailed dispersion mechanism. The conformation of the adsorbed polymer is a major controlling factor in determining the stability and dispersibility of inorganic (nano)particles. In general, adsorbed polymers have three possible segments: (a) an interfacial segment (train), (b) a segment attached to the train at both ends (loop), and (c) a segment attached to the train at one end (tail) [32]. Effective stabilization requires high coverage, effective anchoring, elongated tails (and possibly loops), and a favorable solvent environment for the segments to tail and/or loop. Our polymer design of FGCP Poly**1** allows systematic variation of the lengths of the trains, loops, and tails, and the adsorption conformation and thickness of the adsorption layer can be controlled. Thus, adhesive moiety **1** acts as the train parts, and the length of the loop parts can be manipulated by the composition ratio of adhesive moiety **1** in Poly**4**. The length of the tail parts can be controlled by *D*_p_ of Poly**3**. That is, when the composition ratio of adhesive moiety **1** in the Poly**4** segment is low, Poly**1** has a dominant loop conformation. Conversely, when the composition ratio of adhesive moiety **1** in the Poly**4** segment is high, one end adsorbs to adhesive moiety **1**, and the remaining portion behaves as a tail in a diblock polymer conformation. As a result, the viscosity decreases exponentially as the composition ratio of adhesive moiety **1** increases, in other words, as the Poly**1** copolymer becomes more diblock in nature, as shown in Figure 4B. This result suggests that diblock polymers with **1** at one end are most suitable for the molecular design of BaTiO_3_ dispersants. Furthermore, the present method is also an effective way to efficiently search for the most suitable molecular design of a dispersant consisting of an anchor moiety and a soluble polymer moiety.

## 4. Conclusions

In the present study, we investigated how the monomer position and distribution of the adhesive component of a polymer affect its adhesive strength and dispersion capability to develop an adhesive material that mimics the behavior of a mussel’s foot. We synthesized various FGCPs comprising EHMA by carefully controlling the position and distribution of the gallic-acid-based adhesive component. A copolymer consisting of an EHMA homopolymer as the first domain and a random copolymer of EHMA/**1** as the second domain showed the highest adhesion strength. A block copolymer consisting of Poly**3** and Poly**4** showed the greatest dispersion ability with regard to BaTiO_3_ nanoparticles. The results of the present research demonstrate the importance of the position and distribution of the monomers in the development of functional polymer materials. Because the adhesion and dispersion properties of Poly**1** are comparable with those obtained in previous studies, we are considering potential applications of Poly**1** as an adhesive and a dispersant. Furthermore, because the search for optimal sequences proposed in this paper is not limited to biomimetic materials, such as l-Dopa, FGCPs could facilitate the development of various functional polymer materials.

## Figures and Tables

**Table 1:** Physical Properties of the Gallic-Acid-Bearing Polymers.

Polymer	EHMA:1^*a*^	*M*_n_ (×10^4^) *^b, c^*	*Đ ^b, c^*
Poly**1a**	93.5:6.5	5.1	1.1
Poly**1b**	94.4:5.6	4.3	1.2
Poly**1c**	93.8:6.2	4.3	1.1
Poly**1d**	93.7:6.3	5	1.1
Poly**1e**	95.6:4.4	4.1	1.1
Poly**3**	100:0	3.1	1.2

*^a^* Ratio of EHMA to **1** in the copolymer, as determined by ^1^H NMR. *^b^* Results for triethylsilyl-protected polymers. *^c^* Determined by gel permeation chromatography against poly(methyl methacrylate) standards at 40 °C.

**Table 2:** Adhesive Strength of the Gallic-Acid-Bearing Polymers.

Polymer	*D*_p_ of Poly3	1 in Poly4 (mol%)	Adhesive Strength (MPa)
Poly1a	0	7	7.9
Poly1b	49	9	9.8
Poly1c	98	14	8.6
Poly1d	147	26	4.1
Poly1e	186	100	2.9
Poly3	200	0	2.8

## Data Availability

Not applicable.

## Supplementary Materials

The following supporting information can be downloaded at: https://www.mdpi.com/article/10.3390/ma16010266/s1, Synthesis procedures of Poly**1**–**3**, preparation procedure of a test specimen for the adhesion test, Scheme S1:General synthesis method of a forced gradient copolymer; Figure S1: Preparation of a test specimen; Figure S2: (A) Photograph of LUMiFrac® (LUM GmbH, Germany). (B) Photograph of the measuring chamber; Figure S3: Photograph of the fracture surfaces of adhesion tests of (a) Poly1a, (b)Poly1b, (c)Poly1c, (d)Poly1d, (e)Poly1e and (f)Poly3; Figure S4: GPC profiles of (a) poly**1b**, (b) poly**1c**, (c) poly**1d,** and (d) poly**1e**; Figure S5: ^1^H NMR spectrum of **1’** in CDCl_3_; Figure S6: ^13^C NMR spectrum of **1’** in CDCl_3_**;** Figure S7: ^1^H NMR spectrum of poly**1a** in acetone; Figure S8: ^1^H NMR spectrum of poly**1b** in acetone; Figure S9: ^1^H NMR spectrum of poly**1c** in acetone; Figure S10**:** ^1^H NMR spectrum of poly**1d** in acetone; Figure S11: ^1^H NMR spectrum of poly**1e** in acetone; Figure S12: ^1^H NMR spectrum of poly**3** in CDCl_3_.
