# Supplementary material for: Forced Gradient Copolymer for Rational Design of Mussel-Inspired Adhesives and Dispersants
Source: Materials (Basel). 2022 Dec 27;16(1):266. doi: 10.3390/ma16010266 (PMC9822366; doi:10.3390/ma16010266)
Supplement: Supplementary file 1 [file materials-16-00266-s001.zip › materials-2073960-supplementary.pdf]

Supplementary Information

For

Forced Gradient Copolymer for Rational Design of Mussel-Inspired Adhesives and Dispersants

Takehiro Fujita [1,2], Masami Shuta [1], Mika Mano [1], Shinnosuke Matsumoto [3], Atsushi Nagsawa [3], Akihiro Yamada [3] and Masanobu Naito [1,2,*]

[1]Data-driven Polymer Design Group, Research and Services Division of Materials Data and Integrated System (MaDIS), National Institute for Materials Science (NIMS), 1-2-1 Sengen, Tsukuba, Ibaraki 305-0047, Japan

[2]Program in Materials Science and Engineering, Graduate School of Pure and Applied Sciences, University of Tsukuba, 1-1-1 Tennodai, Tsukuba, Ibaraki 305-8577, Japan

[3]Oleo & Speciality Chemicals Research Lab., NOF CORPORATION, 56 Ohamacho 1, Amagasaki, Hyogo 660-0095, Japan

1. Characterization.

Nuclear magnetic resonance (NMR) spectra were recorded with JEOL ECS-400 (400 MHz; JEOL Ltd., Japan) at room temperature and reported in ppm (δ). ^{1}H NMR spectra referenced to the tetramethylsilane (0.00 ppm). ^{13}C NMR spectra referenced to the tetramethylsilane (0.00 ppm), $CDCl_3$ (77.16 ppm), $(CD_3)_2SO$ (39.52 ppm).Gel permeation chromatography (GPC) was performed on Viscotek TDA302 (Malvern Panalytical Ltd., United Kingdom) equipped with two linearly connected polystyrene (PSt) mixed gel columns (Tosoh corporation, TSKgel GMHHR-H) at 40 °C using RI detectors. THF. The GPC was calibrated with PMMA standard. Adhesive strength was evaluated by centrifugal force using LUMiFrac® produced by LUM. Centrifugal force was applied at 5 N s^{-1} and the adhesive force was calculated from the force at which the adhesive site was dislodged. Viscosity was measured by applying a shear rate to a target sample using a rotating rheometer fitted with a concentric cylinder with a conical tip.

2. Preparation of copolymers.

*Synthesis of **1'**.*

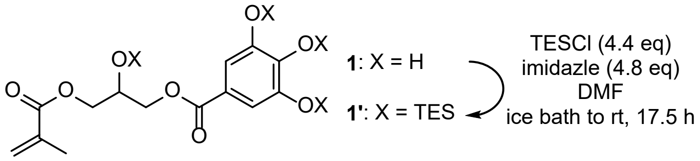

Triethyl silyl chloride (26.2 mL, 155 mmol) was added slowly to a mixture **1** (11.0 g, 35.2 mmol) and imidazole (11.5 g, 169 mmol) in dehydrated DMF (60 mL) under an ice bath, and the resulting solution was stirred at this temperature for 1 h. After more stirring at room temperature for 17.5 h, the resulting mixture was quenched with saturated aqueous $NaHCO_3$ solution, and was extracted with ethyl acetate/hexane (3:1 v/v). The

combined organic layers were washed with saturated aqueous NaCl solution, dried over $MgSO_4$, filtered, and concentrated under reduced pressure to give the crude mixture. Purification by silica gel chromatography (hexane/ethyl acetate = 98/2) afforded the title compound (18.2 g, 67%). ^1H NMR ($CDCl_3$, 400 MHz): δ = 7.18 (s, 2H), 6.13(s, 1H), 5.59(s, 1H), 4.34(m, 1H), 4.32 (m, 4H) , 1.95(s, 3H), 1.04-0.89(t, 36H), 0.85-0.71(q, 18H), 0.65-0.57(q, 6H) ; ^{13}C NMR ($CDCl_3$, 100 MHz): δ = 167.1, 166.2, 148.1, 143.9, 136.2, 125.8, 121.8, 114.8, 68.4, 65.9, 65.7, 1834, 6.8, 6.6, 5.3, 5.1, 4.9.

*Synthesis of the random copolymer Poly**1a**.*

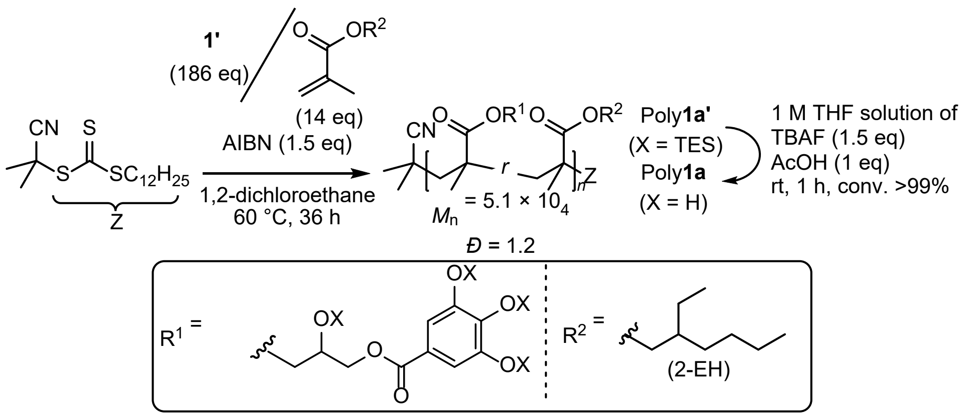

2-Cyano-2-propyl Dodecyl Trithiocarbonate (33 μL, 0.092 mmol), EHMA (3.80 mL, 12.9 mmol), **1'** (1.0 g, 1.30 mmol), and AIBN (15.2 mg, 0.093 mmol) were heated at 60 °C for 12 h. Dichloroethane (6.0 mL) and AIBN (7.6 mg, 0.047 mmol) were added to the reaction mixture, and stirred at 60 °C for 24 h. A small portion of the reaction mixture was taken, and the conversion of the monomers (EHMA:>99% and **1'**:>99%), M_n (5.1 × 10^4), and $Đ$ (1.2) were determined by using ^1H NMR or GPC, respectively. Tetrabutylammonium fluoride solution in THF (1.0 M, 1.6 mL) and acetic acid (xx mL, xx mmol) were added to the reaction mixture, and stirred for 1 h at room temperature for deprotection of triethyl silyl group. The resulting mixture was was poured into vigorously

stirred methanol (1000 mL). The product collected by suction filtration was dried under reduced pressure at 60 °C for 15 h to give poly**1a** (3.6 g, 94%).

*Synthesis of the block copolymer poly**1b**.*

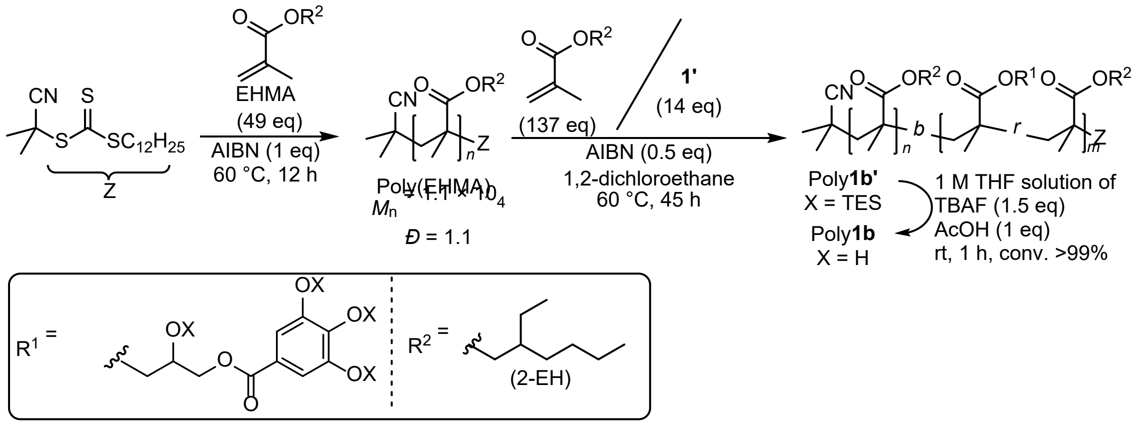

2-Cyano-2-propyl dodecyl trithiocarbonate (33 μL, 0.092 mmol), EHMA (1.0 mL, 4.5 mmol), and AIBN (15.2 mg, 0.093 mmol) were heated at 60 °C for 12 h. A small portion of the reaction mixture was taken, and the conversion of EHMA (94%), M_n (1.1 × 10^4), and Đ (1.1) were determined by using ^{1}H NMR or GPC, respectively. The crude mixture of the first block was used to the next step without further purification.

EHMA (2.8 mL, 12.8 mmol), **1'** (1.0 g, 1.30 mmol), AIBN (7.6 mg, 0.047 mmol) and dichloroethane (1.1 mL) were added to the crude mixture, and the resulting solution was heated at 60 °C for 45 h. A small portion of the reaction mixture was taken, and the conversion of the monomers (EHMA:99% and **1'**:97%), M_n (4.3 × 10^4), and Đ (1.2) were determined by using ^{1}H NMR or GPC, respectively. Dichloroethane (10 mL), tetrabutylammonium fluoride solution in THF (1.0 M, 8.0 mL, 7.8 mmol) and acetic acid (0.30 mL, 5.2 mmol) were added to the reaction mixture, and stirred for 1 h at room temperature. The resulting mixture was poured into vigorously stirred methanol (1000 mL). The product collected by suction filtration was dried under reduced pressure at 60 °C

for 15 h to give poly**1b** (3.7 g, 97%).

*Synthesis of the block copolymer poly**1c**.*

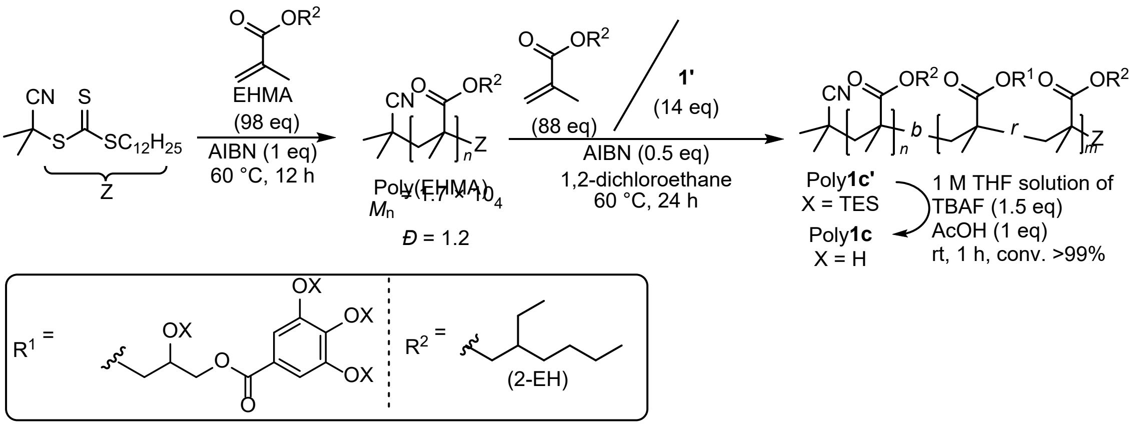

2-Cyano-2-propyl dodecyl trithiocarbonate (33 μL, 0.092 mmol), EHMA (2.0 mL, 9.1 mmol), and AIBN (15.2 mg, 0.093 mmol) were heated at 60 °C for 12 h. A small portion of the reaction mixture was taken, and the conversion of EHMA (97%), M_n (1.7 × 10^4), and $Đ$ (1.2) were determined by using ^{1}H NMR or GPC, respectively. The crude mixture of the first block was used to the next step without further purification.

EHMA (1.8 mL, 8.2 mmol), **1'** (1.0 g, 1.30 mmol), AIBN (7.6 mg, 0.047 mmol) and dichloroethane (2 mL) were added to the crude mixture, and the resulting solution was heated at 60 °C for 24 h. A small portion of the reaction mixture was taken, and the conversion of the monomers (EHMA:>99% and **1'**:>99%), M_n (4.3 × 10^4), and $Đ$ (1.1) were determined by using ^{1}H NMR or GPC, respectively. Dichloroethane (10 mL), tetrabutylammonium fluoride solution in THF (1.0 M, 8.0 mL, 7.8 mmol) and acetic acid (0.30 mL, 5.2 mmol) were added to the reaction mixture, and stirred for 1 h at room temperature. The resulting mixture was poured into vigorously stirred methanol (1000 mL). The product collected by suction filtration was dried under reduced pressure at 60 °C for 15 h to give poly**1c** (3.4 g, 89%).

*Synthesis of the block copolymer poly**1d**.*

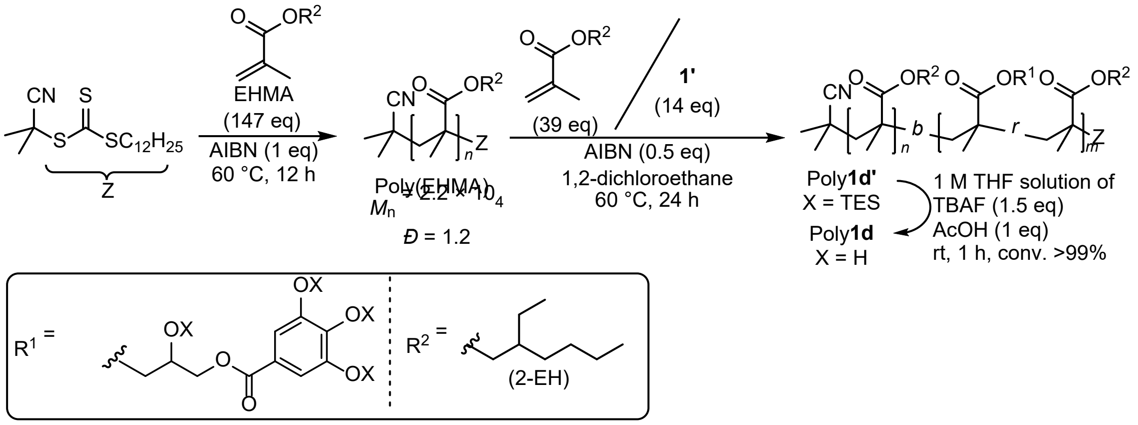

2-Cyano-2-propyl dodecyl trithiocarbonate (33 μL, 0.092 mmol), EHMA (3.0 mL, 13.7 mmol), and AIBN (15.2 mg, 0.093 mmol) were heated at 60 °C for 12 h. A small portion of the reaction mixture was taken, and the conversion of EHMA (99%), M_n (2.2 × 10^4), and *Đ* (1.2) were determined by using ^{1}H NMR or GPC, respectively. The crude mixture of the first block was used to the next step without further purification.

EHMA (0.8 mL, 3.6 mmol), **1'** (1.0 g, 1.30 mmol), AIBN (7.6 mg, 0.047 mmol) and dichloroethane (2.5 mL) were added to the crude mixture, and the resulting solution was heated at 60 °C for 24 h. A small portion of the reaction mixture was taken, and the conversion of the monomers (EHMA:>99% and **1'**:>99%), M_n (5.0 × 10^4), and *Đ* (1.1) were determined by using ^{1}H NMR or GPC, respectively. Dichloroethane (10 mL), tetrabutylammonium fluoride solution in THF (1.0 M, 8.0 mL, 7.8 mmol) and acetic acid (0.30 mL, 5.2 mmol) were added to the reaction mixture, and stirred for 1 h at room temperature. The resulting mixture was poured into vigorously stirred methanol (1000 mL). The product collected by suction filtration was dried under reduced pressure at 60 °C for 15 h to give poly**1d** (3.4 g, 89%).

*Synthesis of the block copolymer poly**1e**.*

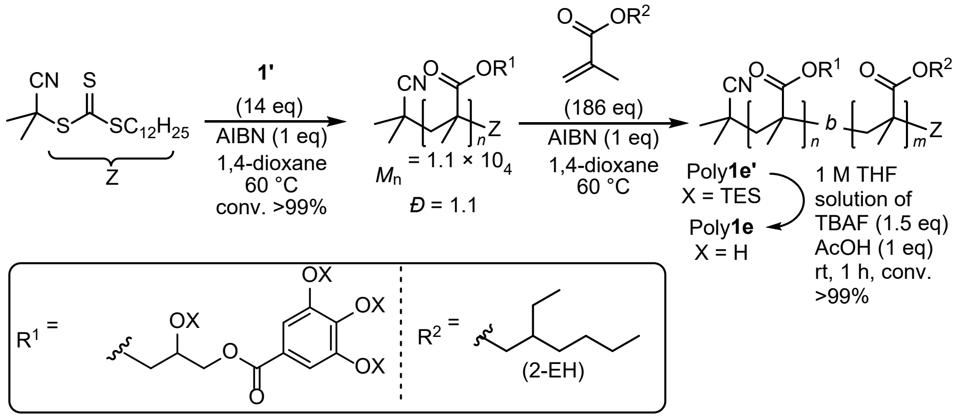

2-Cyano-2-propyl dodecyl trithiocarbonate (33 μL, 0.092 mmol), **1'** (1.0 g, 1.30 mmol), and AIBN (15.2 mg, 0.093 mmol) were heated at 60 °C for 12 h. A small portion of the reaction mixture was taken, and the conversion of **1'** (99%), M_n (1.1 × 10^4), and *Đ* (1.1) were determined by using ^{1}H NMR or GPC, respectively. The crude mixture of the first block was used to the next step without further purification.

EHMA (3.80 mL, 12.9 mmol), AIBN (7.6 mg, 0.047 mmol) and dichloroethane (0.9 mL) were added to the crude mixture, and the resulting solution was heated at 60 °C for 69 h. A small portion of the reaction mixture was taken, and the conversion of EHMA (99%), M_n (4.1 × 10^4), and *Đ* (1.2) were determined by using ^{1}H NMR or GPC, respectively. Dichloroethane (10 mL), tetrabutylammonium fluoride solution in THF (1.0 M, 8.0 mL, 7.8 mmol) and acetic acid (0.30 mL, 5.2 mmol) were added to the reaction mixture, and stirred for 1 h at room temperature. The resulting mixture was poured into vigorously stirred methanol (1000 mL). The product collected by suction filtration was dried under reduced pressure at 60 °C for 15 h to give poly**1e** (3.3 g, 86%).

*Synthesis of poly**2**.*[1,2]

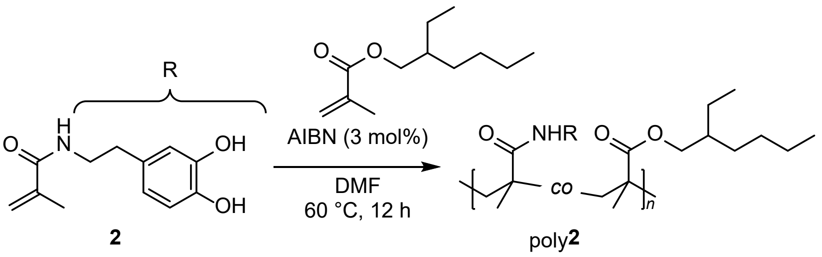

2 (0.7 g, 3.2 mmol), EHMA (3.5 mL, 15.4 mmol), AIBN (80.4 mg, 0.49 mmol) and DMF (1.9 mL) were heated at 60 °C for 12 h. The reaction mixture was dissolved to chloroform/acetone (1vol.%/1vol.%, 110 mL) and the resulting mixture was poured into vigorously stirred methanol (2000 mL). The product collected by suction filtration was dried under reduced pressure at 80 °C for 15 h to give poly**2** (2.7 g). GPC and ^{1}H NMR measurements could not be performed due to the low solubility of the resulting polymer.

*Synthesis of poly**3**.*

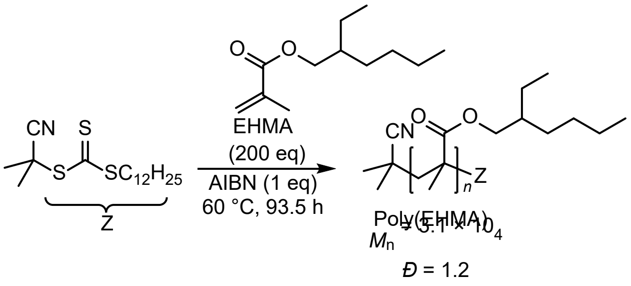

2-Cyano-2-propyl Dodecyl Trithiocarbonate (33 μL, 0.092 mmol), EHMA (4.20 mL, 18.6 mmol), and AIBN (15.2 mg, 0.093 mmol) were heated at 60 °C for 93.593.5 h. A small portion of the reaction mixture was taken, and the conversion of EHMA (93%), M_n (3.1×10^4), and $Đ$ (1.2) were determined by using ^{1}H NMR or GPC, respectively. The resulting mixture was poured into vigorously stirred methanol (1000 mL). The product collected by suction filtration was dried under reduced pressure at 60 °C for 15 h to give poly**3** (3.5 g, 93%).

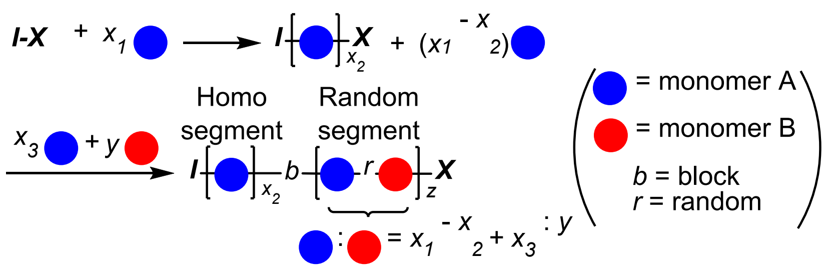

Scheme S1. General synthesis method of a forced gradient copolymer.

3. Preparation of a test specimen for adhesion test (Figure S1).

A copolymer was dissolved in chloroform or THF to concentration of 125 g L^{-1}, and the 75 μL of resulting solution was spread over an aluminum stamp (Adhesive area = 78.5 mm^2). A solvent on a stamp was vapored at room temperature and a resulting stamp was attached to weight (18.3 g). A test stamp was overlapped with center of an aluminum plate (20 mm × 20 mm × 2 mm), and it was cured at 80 or120 °C for 1 h. The resulting test specimen was allowed to cool for 1 h and used to a tensile strength test.

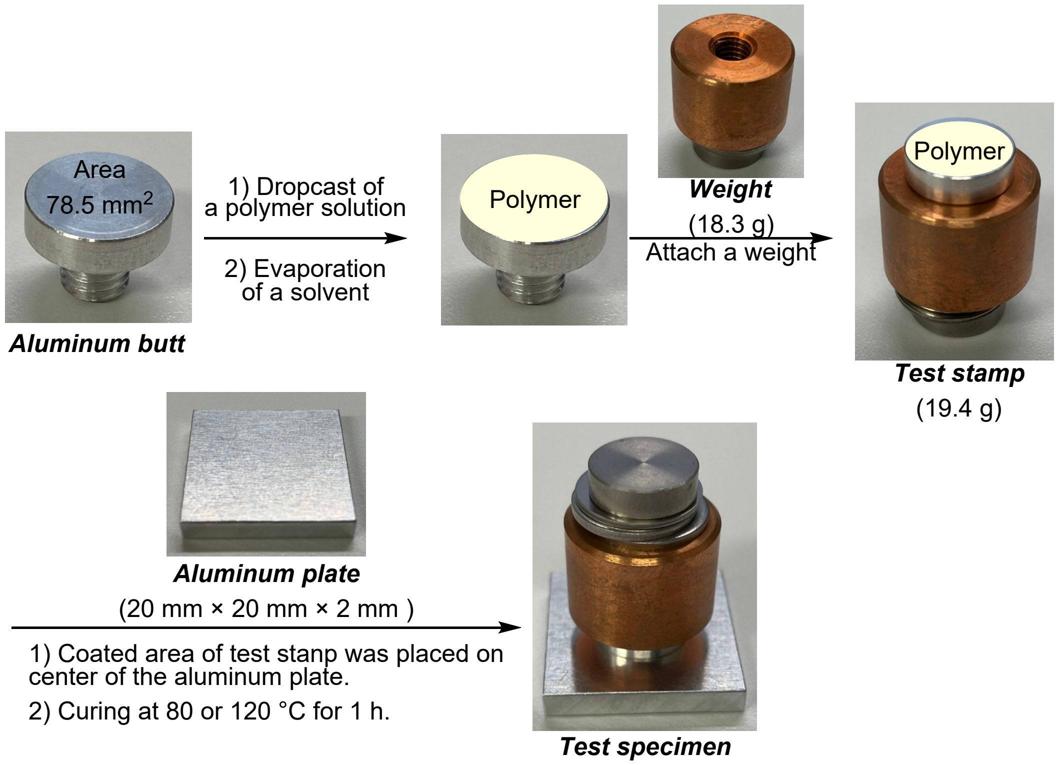

Figure S1. Preparation of a test specimen.

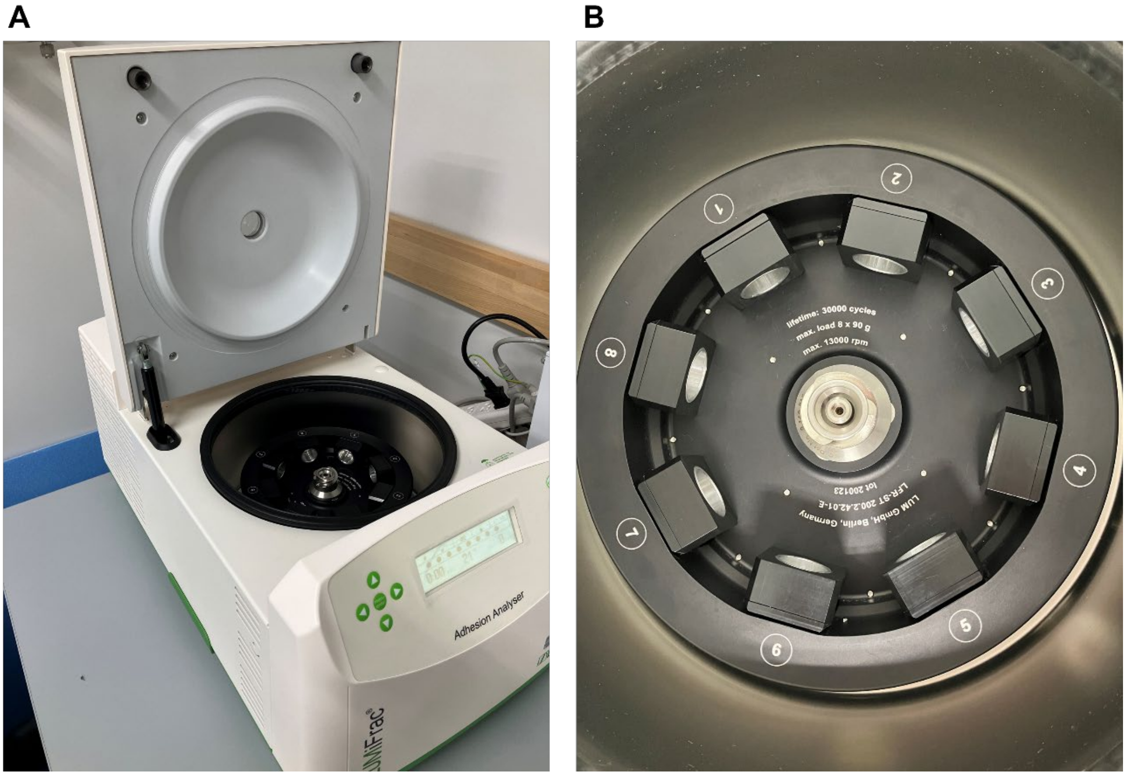

Figure S2. (A) Photograph of LUMiFrac® (LUM GmbH, Germany). (B) Photograph of the measuring chamber.

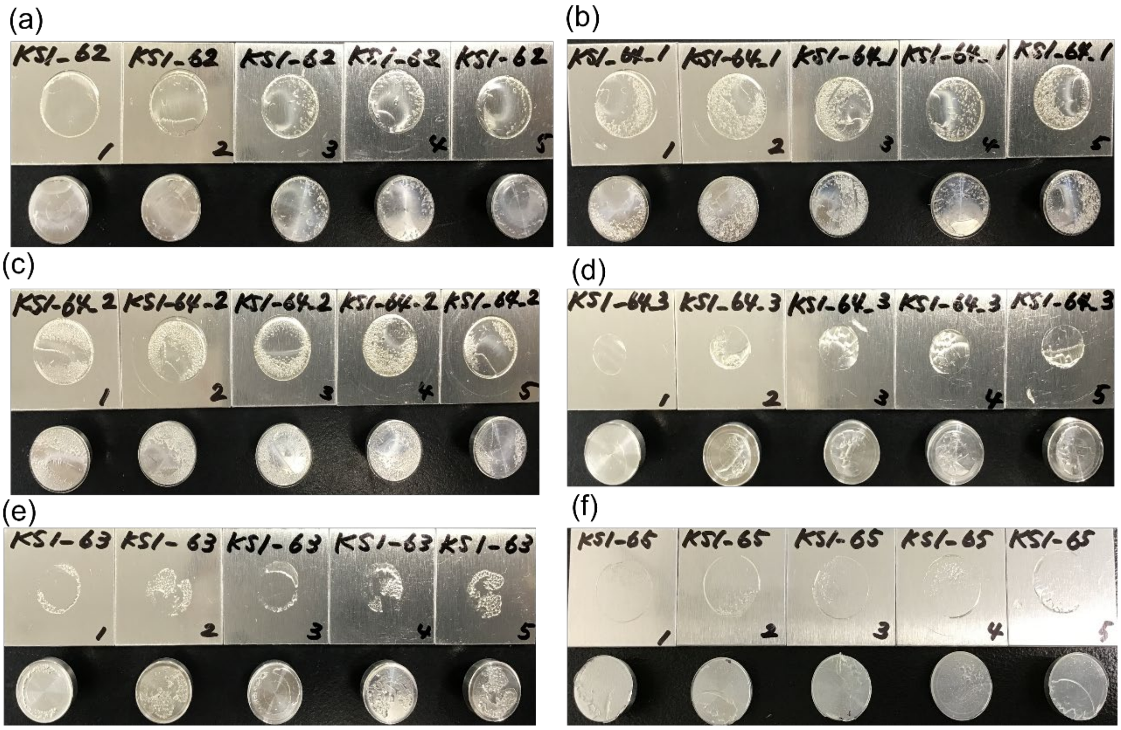

Figure S3. Photograph of the fracture surfaces of adhesion tests of (a) Poly<u>**1**</u>**a**, (b)Poly<u>**1**</u>**b**, (c)Poly<u>**1**</u>**c**, (d)Poly<u>**1**</u>**d**, (e)Poly<u>**1**</u>**e** and (f)Poly<u>**3**</u>.

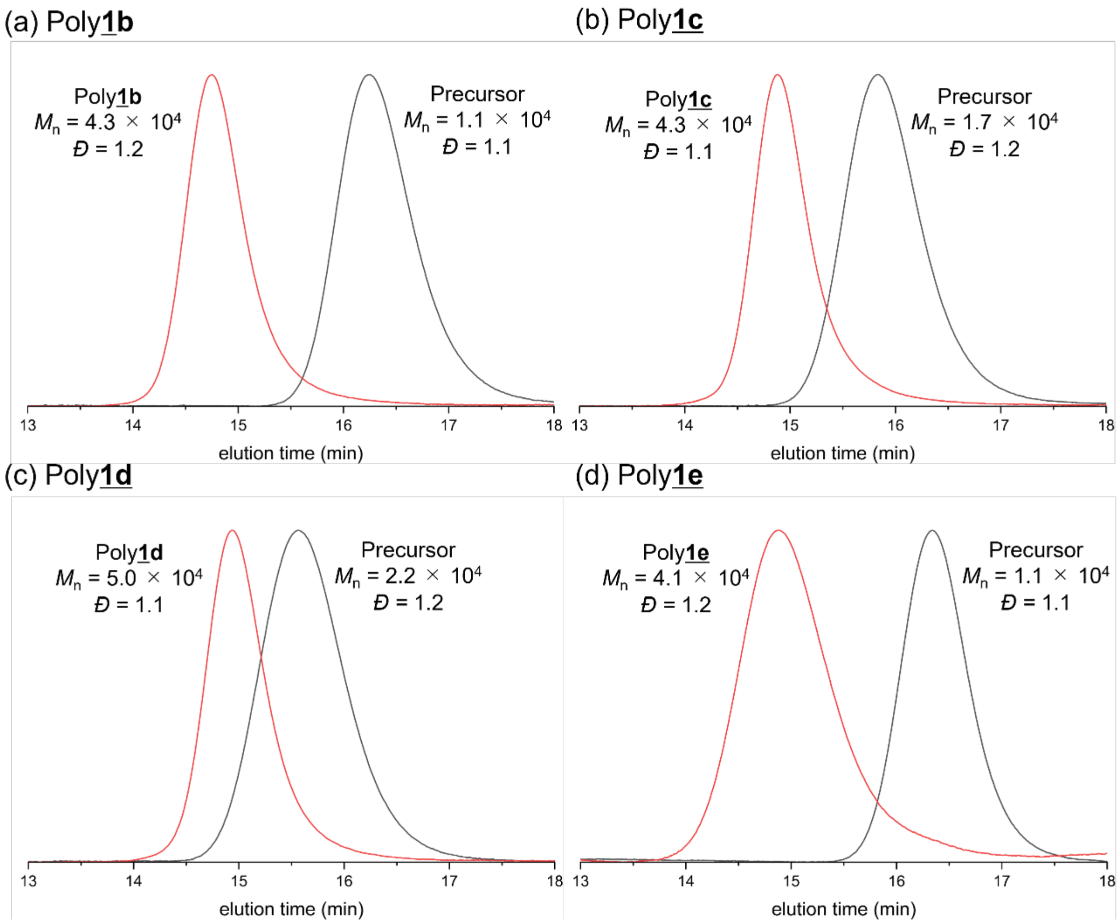

Figure S4. GPC profiles of (a) poly**1b**, (b) poly**1c**, (c) poly**1d** and (d) poly**1e**.

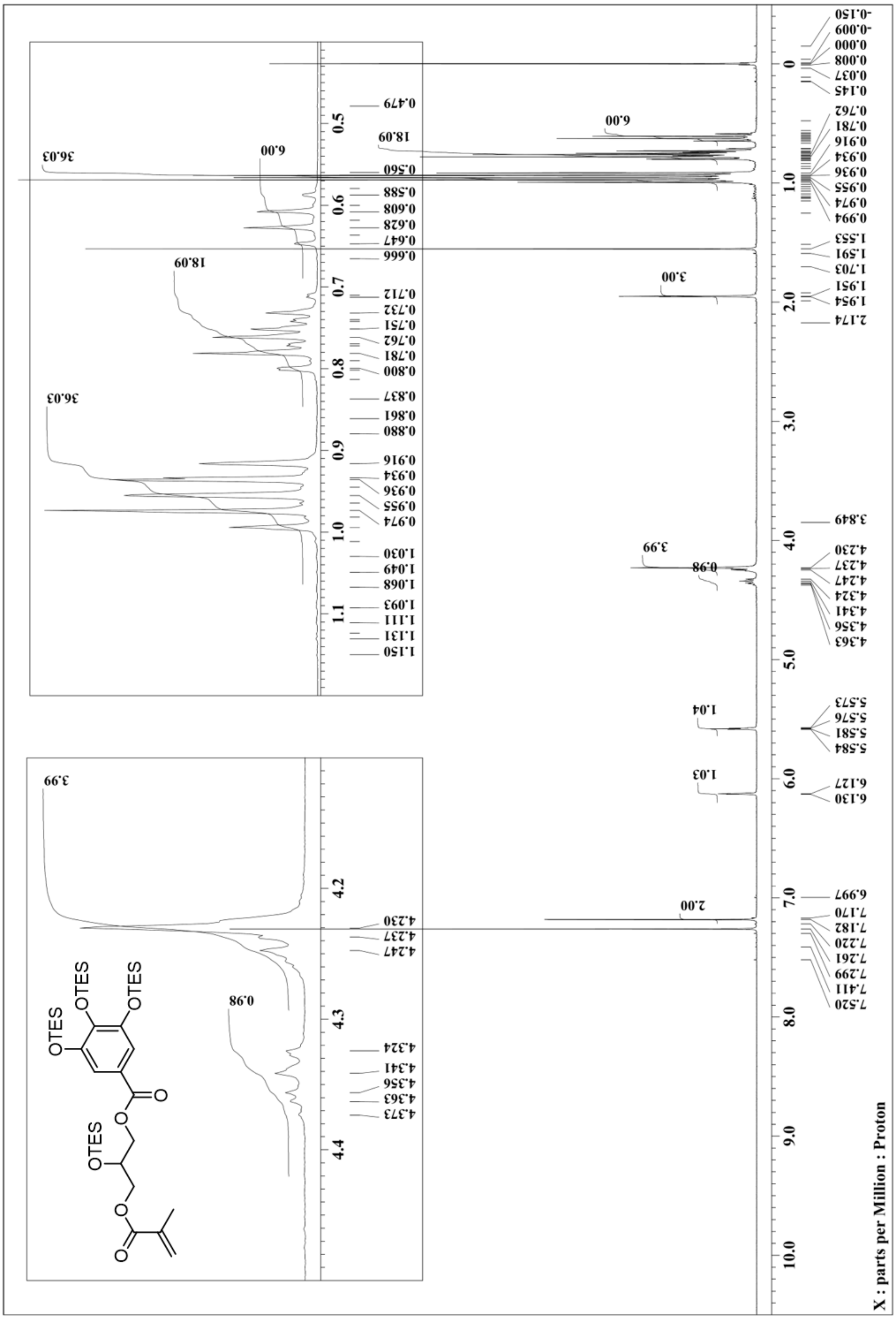

Figure S5. ^{1}H NMR spectrum of **1'** in $CDCl_3$.

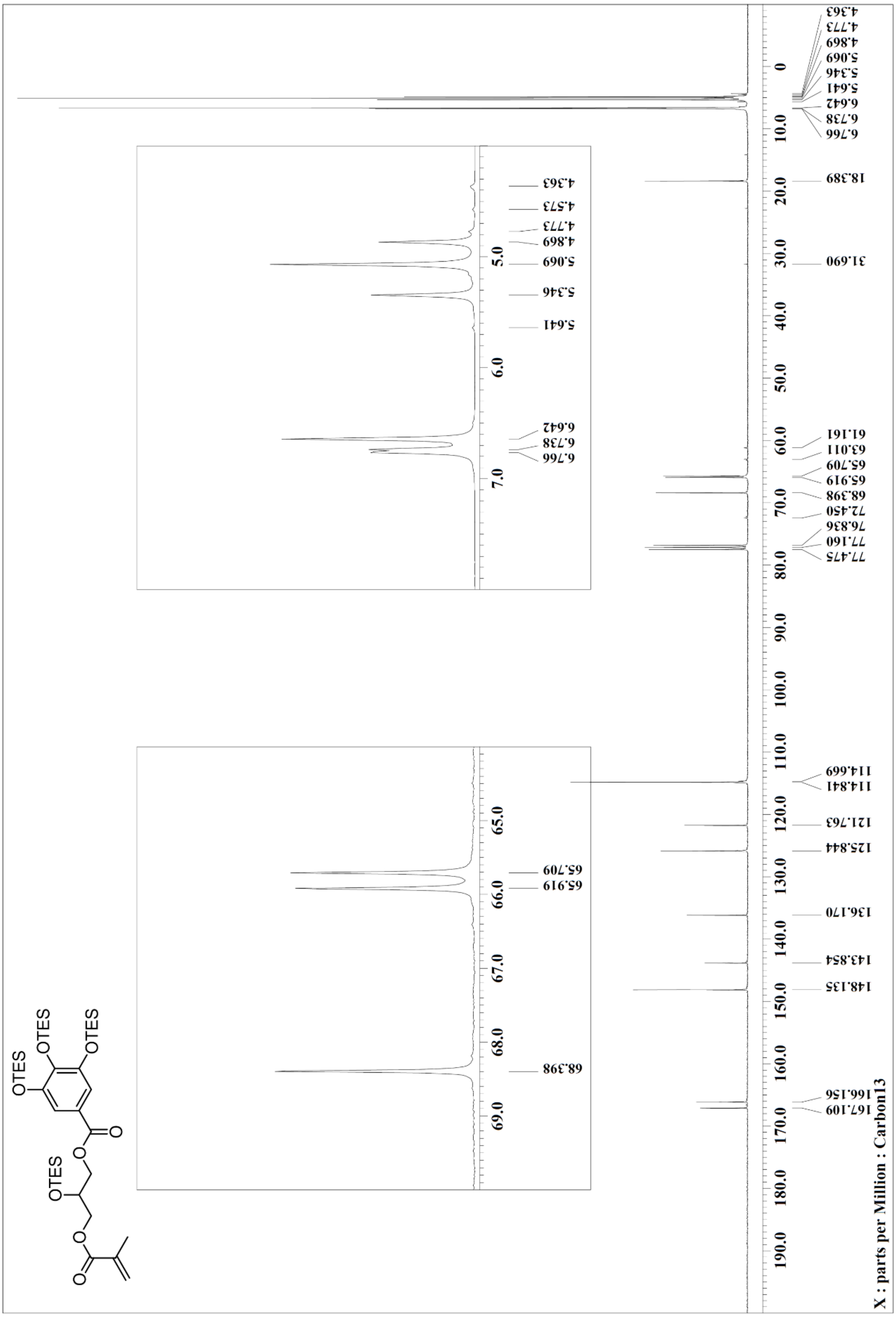

Figure S6. ^{13}C NMR spectrum of **1'** in $CDCl_3$.

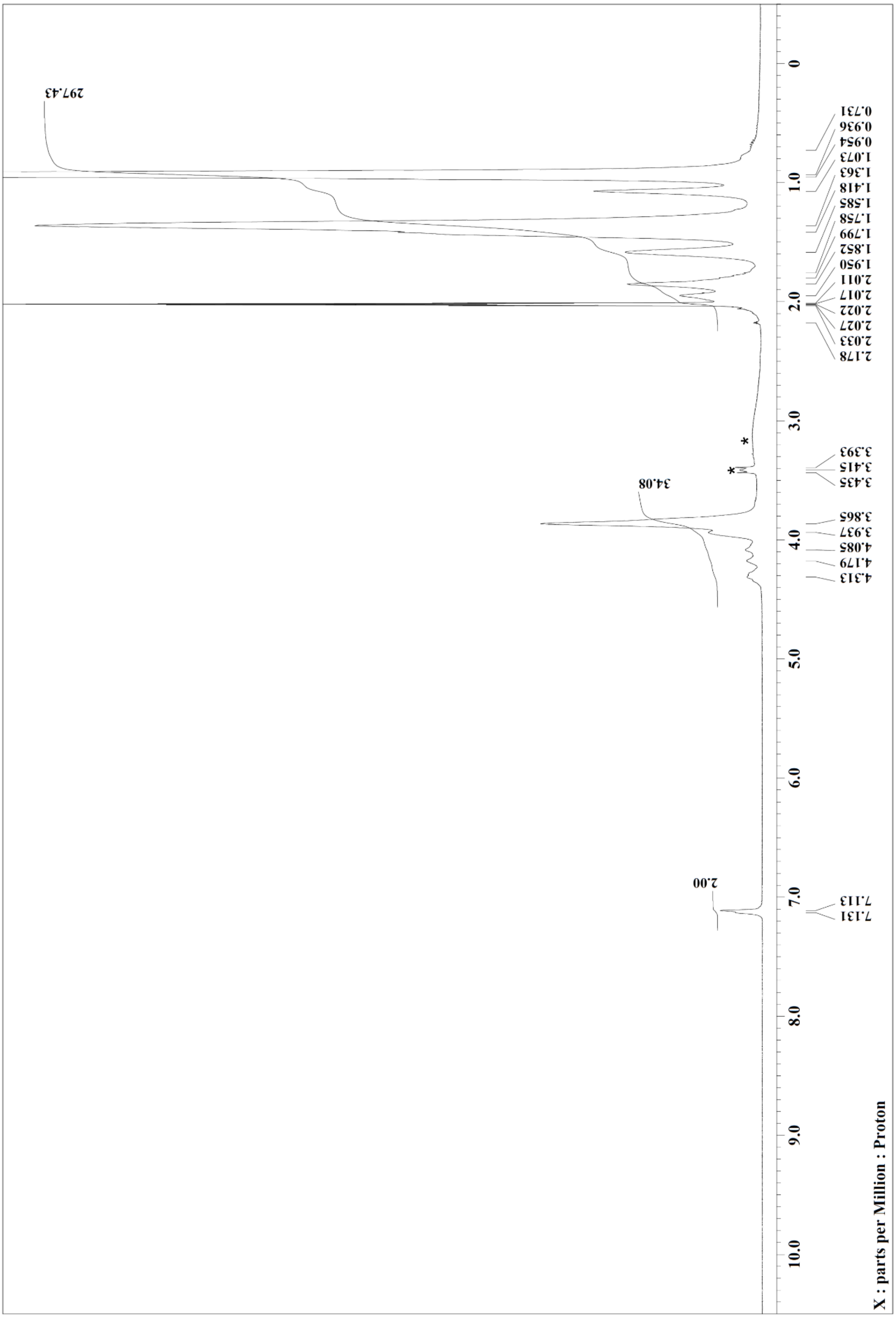

Figure S7. ^{1}H NMR spectrum of poly**1a** in acetone.

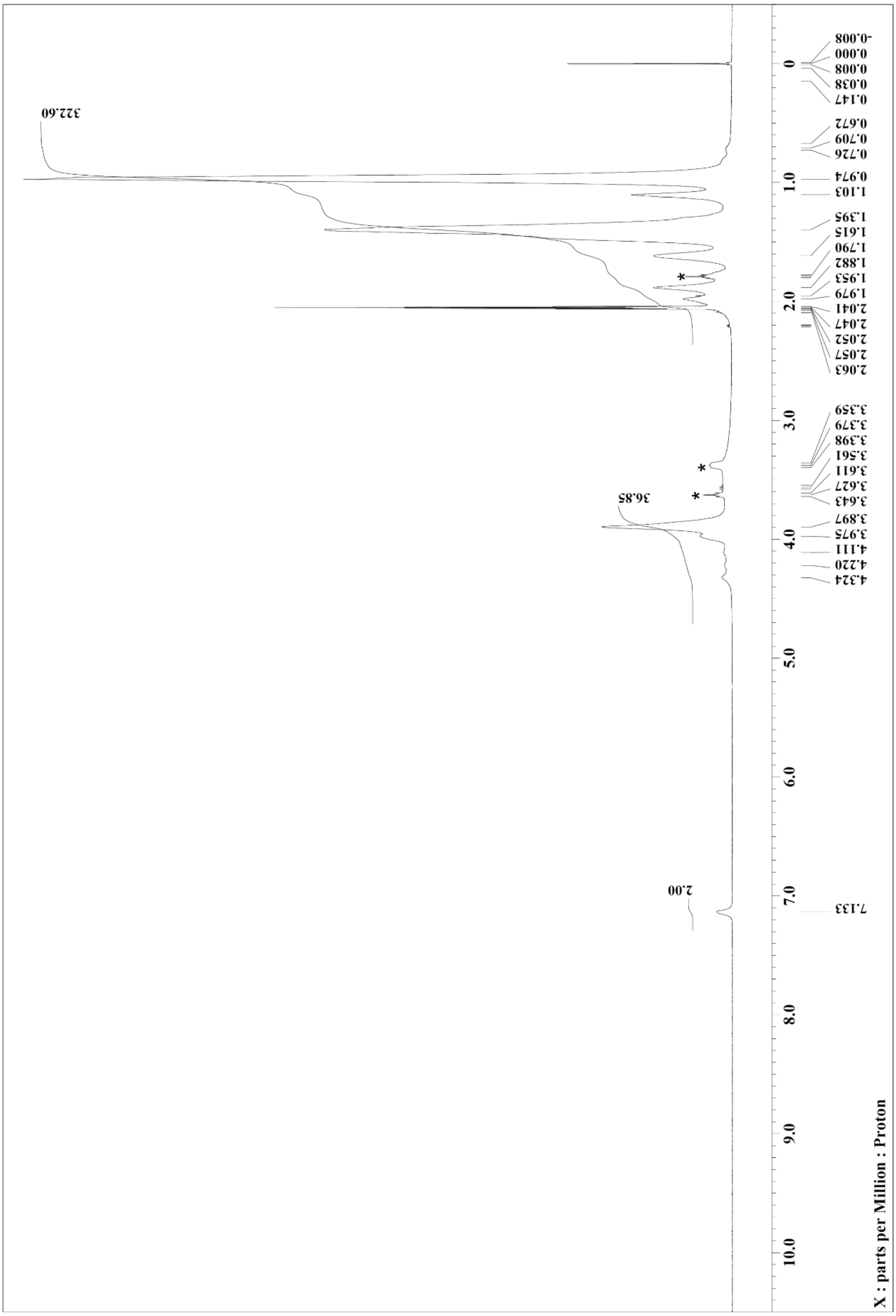

Figure S8. ^{1}H NMR spectrum of poly**1b** in acetone.

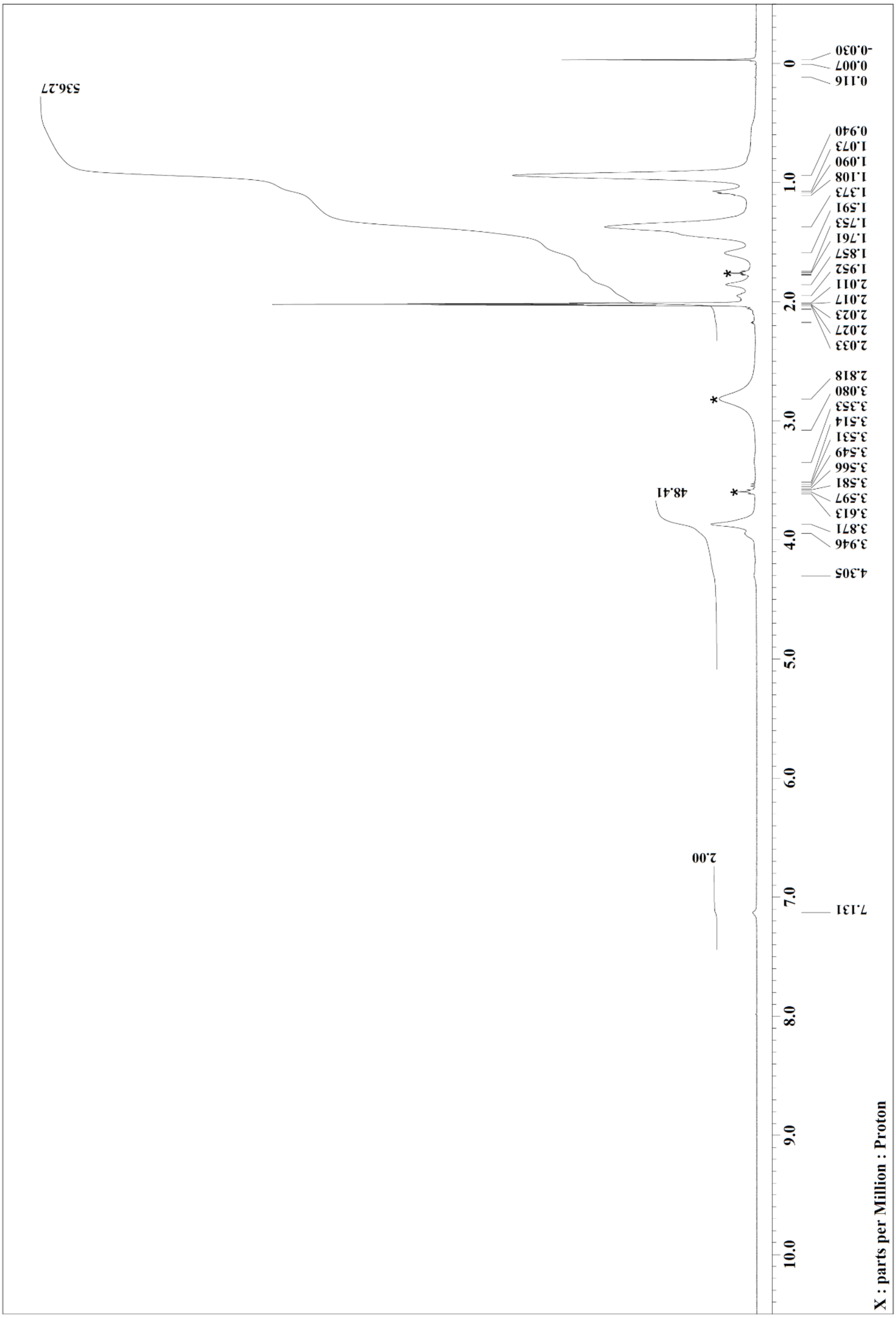

Figure S9. ^{1}H NMR spectrum of poly**1c** in acetone.

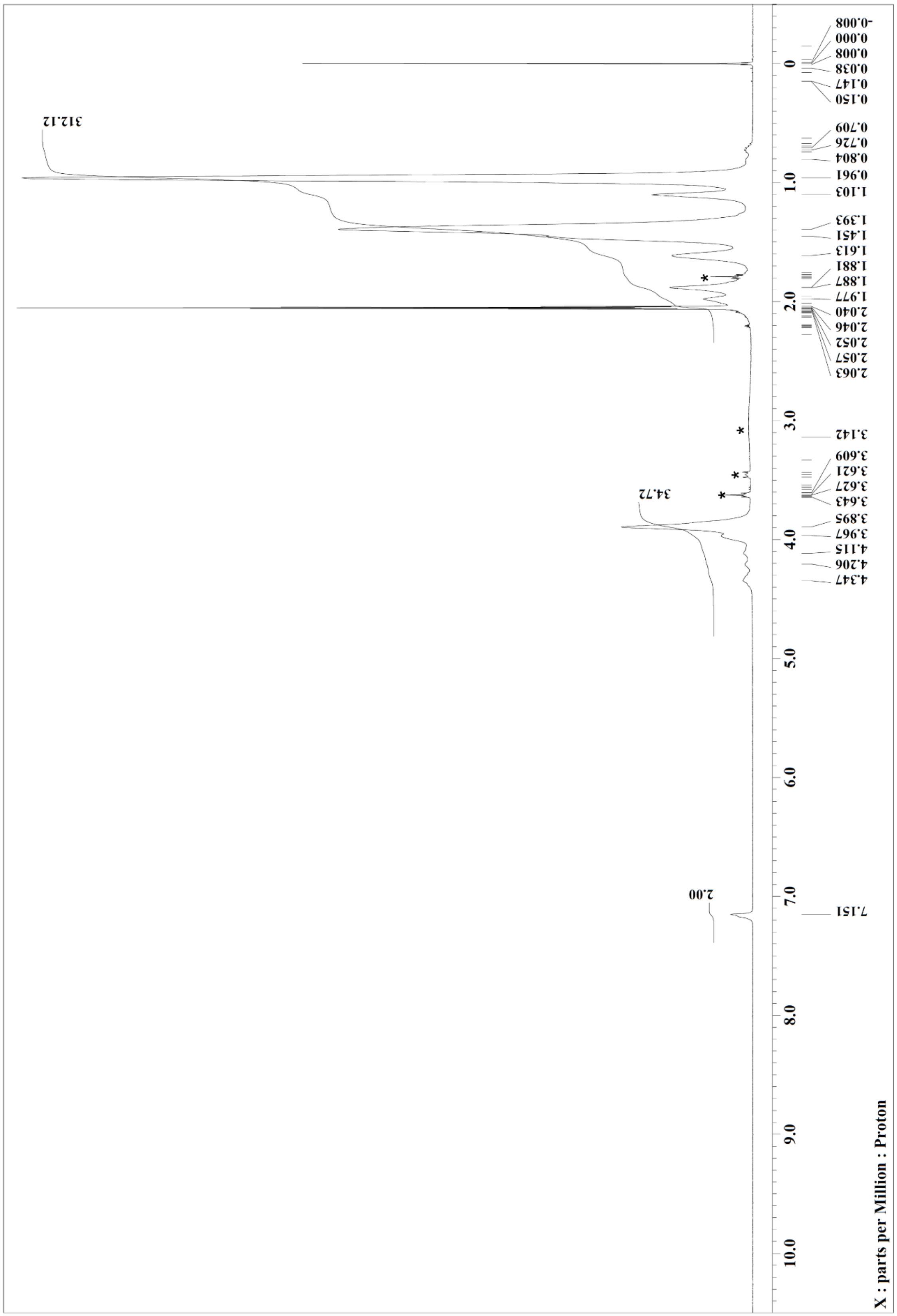

Figure S10. ^{1}H NMR spectrum of poly**1d** in acetone.

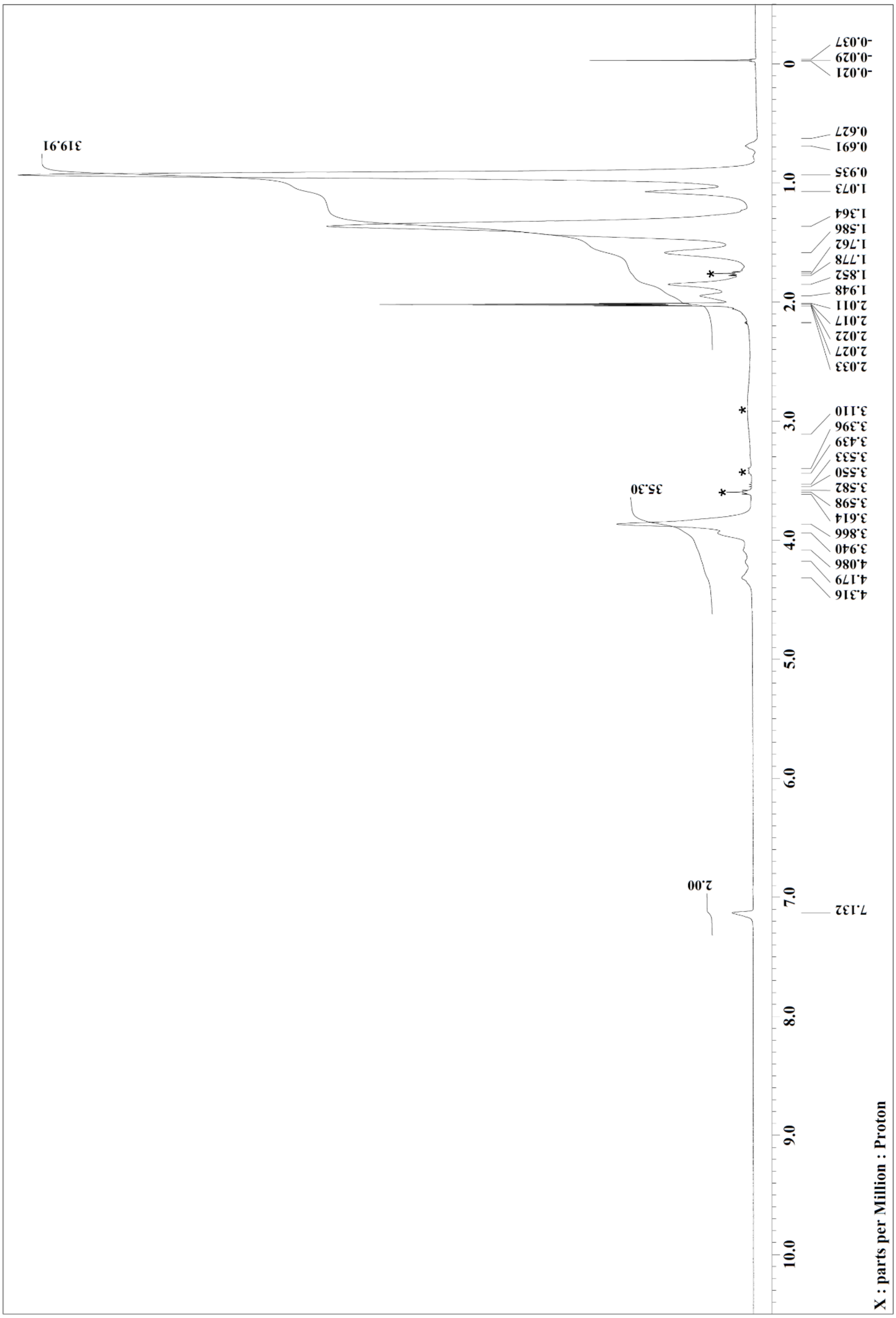

Figure S11. ^{1}H NMR spectrum of poly**1e** in acetone.

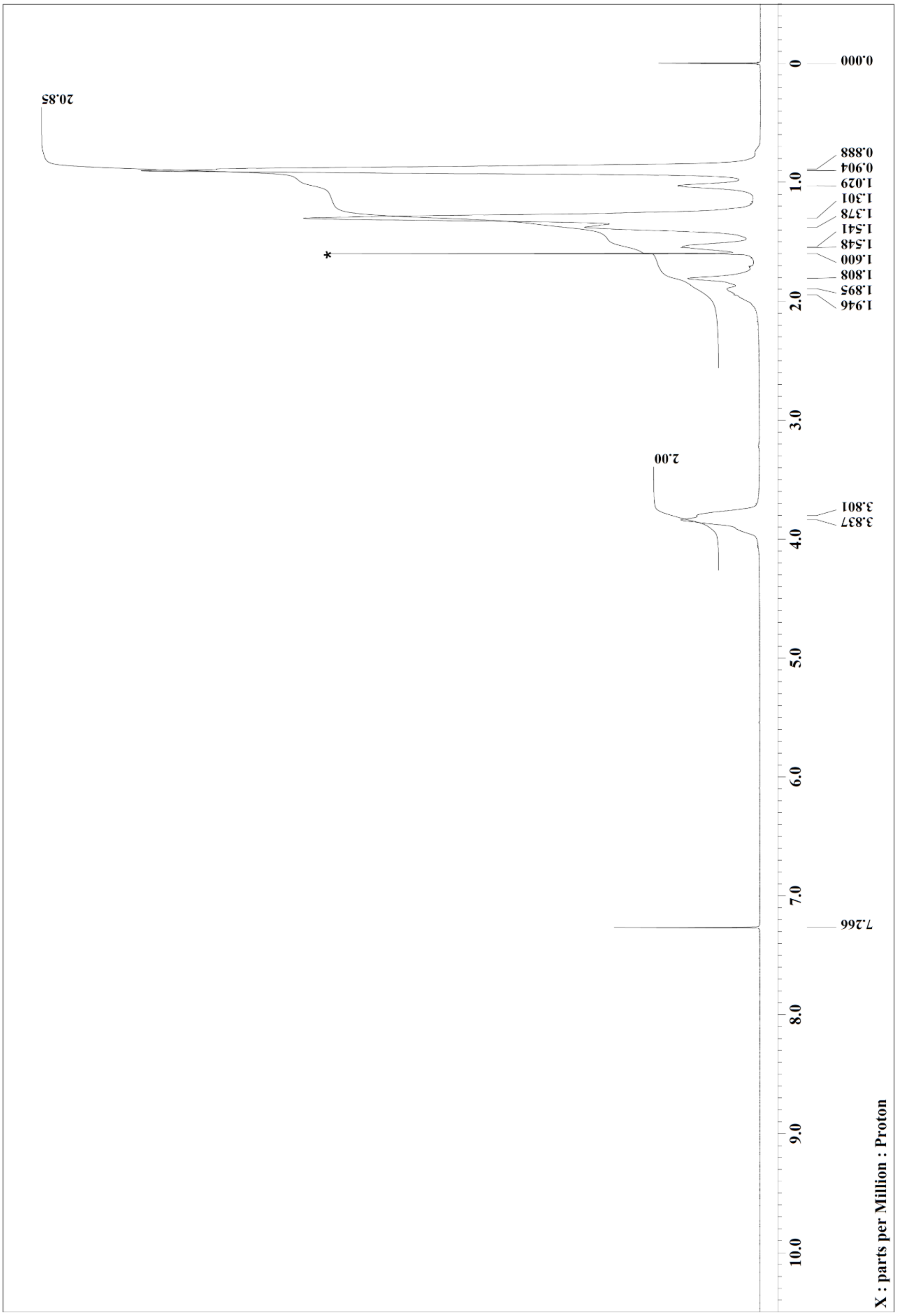

Figure S12. ^{1}H NMR spectrum of poly**3** in $CDCl_3$.

Reference

1. Payra, D.; Naito, M.; Fujii, Y.; Yamada, N. L.; Hiromoto, S.; Singh, A. Bioinspired adhesive polymer coatings for efficient and versatile corrosion resistance. *RSC Adv.* **2015,** *5*, 15977–15984.
2. Payra, D.; Fujii, Y.; Das, S.; Takaishi, J.; Naito, M. Rational design of a biomimetic glue with tunable strength and ductility. *Polym. Chem.* **2017,** *8*, 1654–1663.
